# Identification of antibodies induced by immunization with the syphilis vaccine candidate Tp0751

**DOI:** 10.1016/j.vaccine.2025.126804

**Published:** 2025-02-04

**Authors:** Francesca Urselli, Alloysius Gomez, Matthew D. Gray, Caroline E. Cameron, Justin J. Taylor

**Affiliations:** a Fred Hutchinson Cancer Center, Seattle, WA, USA; b Department of Biochemistry and Microbiology, University of Victoria, Victoria, BC, Canada; c Division of Allergy & Infectious Diseases, Department of Medicine, University of Washington, Seattle, WA, USA; d Beirne B. Carter Center for Immunology Research, University of Virginia, Charlottesville, VA, USA; e Division of Infectious Diseases & International Health, Department of Medicine, University of Virginia, Charlottesville, VA, USA; f Department of Microbiology, Immunology, and Cancer Biology, University of Virginia, Charlottesville, VA, USA

**Keywords:** Antibodies, Syphilis, Vaccine, Tp0751, B cell, Rabbit

## Abstract

The continued and increasing prevalence of syphilis worldwide highlights the need for an effective syphilis vaccine to complement public health measures. Previous work demonstrated that immunization of the rabbit animal model with vaccine candidates derived from the *T. pallidum* endothelial cell adhesin Tp0751 could reduce dissemination of *T. pallidum* to lymph nodes. In those studies, a proportion of animals exhibited complete inhibition of treponemal dissemination and others exhibited partial or no inhibition of treponemal dissemination, consistent with results expected from an outbred animal model. In the current study we further characterized the Tp0751-specific antibody response in immunized animals that showed inhibition of *T. pallidum* dissemination. To do this, we generated Tp0751 tetramers to identify Tp0751-specific B cells before and after immunization. Using this approach, we found a robust expansion of Tp0751-specific B cells in the blood and spleens of immunized animals compared to unimmunized control animals. Ten antibodies from Tp0751-immunized rabbits were cloned and binding to specific structural regions of the Tp0751 protein was assessed using epitope mapping assays and structural modeling. Importantly, nine out of the ten antibodies cloned from Tp0751 tetramer-binding B cells were able to significantly inhibit *T. pallidum* attachment to human endothelial cells *in vitro*, including antibodies exhibiting weaker binding to Tp0751. Combined, our results provide a proof-of-principle that Tp0751-based subunit vaccines can stimulate strong B cell responses resulting in the production of antibodies able to inhibit *T. pallidum* attachment to endothelial cells.

## 1. Introduction

While vaccine development typically focuses upon pathogen or toxin neutralization, the inhibition of dissemination could serve as a complementary approach for diseases caused by invasive pathogens, including syphilis. Infectious syphilis is a complex chronic disease characterized by widespread dissemination of the bacterium *Treponema pallidum* during the symptomatic and asymptomatic stages. Stages of the disease include the primary stage which is typified by the infectious ulcerative chancre, followed by a secondary stage with disseminated rash, the asymptomatic latency stage, and in some individuals the late tertiary syphilis stage involving serious neurological and cardiovascular symptoms which can arise decades after initial infection [1,2].

Dissemination of *T. pallidum* occurs early during the primary stage, and the symptoms that arise in subsequent stages are believed to be due to the immune response raised to disseminated treponemes. In addition, oral and anal shedding of *T. pallidum* occurs at all stages of infection, which may indicate a key role for dissemination in syphilis transmission [3-5]. Further, neurosyphilis and congenital syphilis occur due to treponemal dissemination across the blood-brain and placental barriers, respectively.

The *T. pallidum* vascular adhesin Tp0751 is a leading target for a dissemination-inhibiting vaccine since this protein plays a role in treponemal dissemination by mediating bacterial attachment to the vasculature [6-9]. Previously, we found that immunization of rabbits with Tp0751-based vaccines provided a spectrum of protection against dissemination, with some immunized rabbits exhibiting no treponemal dissemination to lymph nodes while others exhibited reduced *T. pallidum* dissemination to lymph nodes [6,10]. While these results are promising, further refinements are required to develop a vaccine candidate that can consistently inhibit *T. pallidum* dissemination in pre-clinical protection trials. Here, we begin to probe the mechanism of *T. pallidum* dissemination inhibition through the identification and characterization of antibodies induced by immunization of rabbits with a Tp0751-based vaccine formulation.

## 2. Material and methods

### 2.1. Animals and single cell suspensions

Six- to 14-week-old C57BL/6 male and female mice were purchased from the Jackson Laboratory and maintained in a specific pathogen-free facility in accordance with Fred Hutchinson Cancer Center Institutional Animal Care and Use Committee approval and National Institutes of Health guidelines. The spleen and inguinal, axillary, brachial, cervical, mesenteric, and periaortic lymph nodes from individual mice were pooled, shredded using forceps, and forced through 100-μm mesh twice to generate filtered single cell suspensions. Cell suspensions were then centrifugated at 300 xg for five minutes at 4 °C and supernatant discarded prior to tetramer enrichment experiments.

Outbred male New Zealand White rabbits (3.0–3.5 kg, Charles River Laboratories, Ontario, Canada for UVic studies) with nonreactive VDRL and FTA-ABS *Treponema paraluiscuniculi* serologies were used for *T. pallidum* propagation and for immunization studies. The *T. pallidum* Nichols strain was propagated by serial rabbit passage as previously described [11]. All rabbits were housed at 18–20 °C and fed antibiotic-free food and water. Studies approved by the local institutional review boards at University of Victoria under biosafety certificate 13170–010 and were conducted in strict accordance with standard accepted principles as set forth by the Canadian Council on Animal Care (CCAC), National Institutes of Health, and the United States Department of Agriculture in facilities accredited by the American Association for the Accreditation of Laboratory Animal Care and the CCAC. Frozen rabbit PBMC and splenocytes were shipped from the University of Victoria on dry ice and stored in the liquid nitrogen until use. Samples were thawed at 37 °C and transferred into fresh warm RPMI containing 20 % fetal bovine serum. Cell suspensions were centrifugated at 300 x g for five minutes at 4 °C and supernatant discarded prior to tetramer enrichment experiments.

### 2.2. Recombinant antigens

Recombinant Tp0897 (TprK) fragment 1 (amino acids 37–273) [12] and Tpr Subfamily I Tp0117 (TprC) Nichols template (amino acids 23–351) [13], were produced and purified as previously described [14]. Soluble Tp0751_24–237_, Tp0751_99–237_, Tp0751_115–237_ were expressed and purified as previously described using affinity chromatography and size exclusion chromatography [15].

### 2.3. Immunization and infectious challenge

Mice were injected subcutaneously in the base of the tail with 5 μg of Tp0751_24–237_ in natural RIBI adjuvant produced by PAI Life Sciences seven days prior to analysis.

As described previously [10], rabbits were first injected with 125 μg of Tp0751_24–237,_ 20 μg of TprK and 20 μg of TprC in natural RIBI adjuvant. Animals subsequently received four booster immunizations every three weeks and a fifth booster twenty-six weeks after the fourth booster containing 125 μg of soluble Tp0751_24–237_, 10 μg of TprK, and 10 μg of TprC in natural RIBI adjuvant. One week after the final immunization, PBMC were isolated from each rabbit and cryopreserved. Three weeks following the final injection, immunized and uninjected control rabbits were challenged intradermally at ten sites on their shaved backs with 10^5^ *T. pallidum* Nichols strain per site, and monitored for lesion development and dissemination. Spleens were harvested 40 days after infection, mechanically dissociated into single cell suspensions and 30 million cells were pelleted by centrifugation at 1000 rpm for 10 min at 5 °C and washed three times with ice cold hanks balanced salt solution without magnesium and calcium (HBSS) with the supernatant discarded after each wash step. After the final wash, freezing media (90 % FBS and 10 % DMSO) was added to the cells and cells were transferred to cryovials and placed into a freezing chamber at −80 °C prior to storage in liquid nitrogen.

### 2.4. Rabbit PBMC isolation

Whole blood was collected in tubes containing the anticoagulant Acid Citrate Dextrose (ThermoFisher Scientific). Four mL of blood was diluted in equal volume of 1× DPBS and then carefully layered over 6 mL of Lympholyte-Mammal cell separation media (Cedarlane, Ontario, Canada) in a 15 mL centrifuge tube. The layered mixture was centrifuged at 1500 x*g* for 30 min at 5 °C. The cells from the interface were carefully removed using a Pasteur pipette and diluted with 3 volumes of PBMC wash buffer (25 mM HEPES and 1 % FBS in HBSS).

PBMC were pelleted by centrifugation at 800 x*g* for 10 min at 5 °C and washed three times in ice cold HBSS with the supernatant was discarded after each wash step. After the final wash freezing media containing 90 % FBS and 10 % DMSO was added to the gently resuspended pellet and PBMC were transferred to cryovials and placed into a freezing chamber at −80 °C prior to storage in liquid nitrogen.

### 2.5. Tp0751_24–237_ tetramer production

Antigen biotinylation and tetramer production was conducted as described previously with minor modifications [16-22]. Briefly, Tp0751_24–237_ was biotinylated using EZ-link Sulfo-NHS-LC-Biotinylation kit (ThermoFisher Scientific) using a 1 to 1.3 molar ratio of biotin to Tp0751_24–237_ and unconjugated biotin was removed by centrifugation using a 3 kDa Amicon Ultra size exclusion column (MilliporeSigma). To determine the average number of biotin molecules bound to each molecule of Tp0751_24–237_, streptavidin-PE (Agilent) was titrated into a fixed amount of biotinylated Tp0751_24–237_ at increasing concentrations and incubated at room temperature for 30 min. Samples were run on an SDS-PAGE gel (ThermoFisher Scientific), transferred to nitrocellulose, and incubated with streptavidin-Alexa Fluor 680 (ThermoFisher Scientific) at a dilution of 1:10,000 to determine the point at which there was excess biotinylated protein available for streptavidin–Alexa Fluor 680 reagent to bind. Biotinylated Tp0751_24–237_ was aliquoted and stored at −80 °C until use. Tetramers were produced fresh the day of use by mixing thawed biotinylated Tp0751_24–237_ with streptavidin-APC (Agilent) and streptavidin-PE at the ratio determined above to fully saturate streptavidin, and incubated for 30 min on ice. Unconjugated Tp0751_24–237_ was removed by centrifugation using a 100 kDa Nanosep centrifugal device (Pall Corporation). Tp0751_24–237_ tetramers were stored at 0.1–3 μM in 1× DPBS at 4 °C for less than 2 h prior to use. Of note, while we have taken great care to minimize the probability of higher order complexes forming due to some individual protein molecules containing multiple biotin molecules binding to different streptavidin molecules, the presence of a low level of higher order complexes is possible in the tetramer preparation used.

Control PE-DyLight650 (PE650) APC-DyLight755 (APC755) tetramers were created by mixing 5-fold molar excess of biotinylated 6xHIS peptide (Genscript) with SA-PE pre-conjugated to DyLight650 NHS ester (ThermoFisher Scientific) or streptavidin-APC pre-conjugated with DyLight755 NHS ester (ThermoFisher Scientific) following the manufacturer’s instructions. On average, PE650 and APC755 contained 4–8 DyLight molecules per PE/APC. The concentration of each tetramer was calculated by measuring the absorbance of APC at 650 nm using the extinction coefficient 0.7 μM^−1^ cm^−1^ or PE at 566 nm using the extinction coefficient 1.96 μM^−1^ cm^−1^. Control tetramers were stored at 0.1–3 μM in 1× DPBS at 4 °C or 0.5× DPBS containing 50 % glycerol at −20 °C prior to use.

#### 2.5.1. Tetramer-binding B cell enrichment and flow cytometry

Cell pellets were resuspended in 0.2 mL ice cold FACS buffer (1× DPBS containing 1 % heat-inactivated newborn calf serum). Each fraction received 2 μg of anti-Fc receptor antibody 2.4G2 (BioXCell), 1 pmol of control APC755 tetramer and 1 pmol of control PEDL650 tetramer before incubation on ice for 5 min. Next, 1 pmol of Tp0751_24–237_ PE tetramer and 1 pmol of Tp0751_24–237_ APC tetramer were added and samples incubated for 25 min on ice. After the incubation, ~15 mL of FACS buffer was added and the samples centrifuged at 300 x*g* for five min at 4 °C. The supernatant was discarded, and the pellet resuspended prior to the addition of 25 μL of anti-PE microbeads and 25 μL of anti-APC microbeads (Miltenyi Biotec). Following incubation for 25 min on ice, 5 mL of FACS buffer was added and the samples were passed over a magnetized LS column (Miltenyi Biotec). The tube and column were washed once with 5 mL FACS buffer and then removed from the magnetic field. Five mL of FACS buffer was forced through the column with a plunger twice to elute column-bound cells. All the cells from the tetramer enriched column-bound fraction and 1/20th - 1/40th of the tetramer depleted column flow through fraction were centrifuged at 300 x*g* for five minutes at 4 °C. The supernatant was discarded, and the pellet resuspended prior to the addition of a 25 μL cocktail containing antibodies targeting surface proteins and a viability dye and incubated for 25 min on ice. For mouse cells the cocktail included 0.1 μg anti-CD38 Alexa Fluor 700 (90, BD Biosciences), 0.2 μg GL7 FITC (GL7, BD Biosciences), 0.4 μg anti-B220 BV786 (RA3-6B2, BioLegend), 0.3 μg anti-CD19 BUV395 (1D3, BD Biosciences), 0.2 μg anti-CD3 BV510 (145-2C11, BD Biosciences), 0.2 μg anti-F4/80 BV510 (BM8, BioLegend), and 0.2 μg anti-Gr-1 BV510 (RB6-8C5, BD Biosciences), and 0.5 μL Fixable Viability Stain 620 (BD Biosciences). For rabbit cells that cocktail included 10 μL anti-IgM FITC (BioRad), 1 μL anti-CD4 FITC (BioRad), 1 μL anti-CD8 FITC (BioRad), 1 μL anti-Ig BV421 (BD Bioscience), and 1 μL Ghost Dye Violet 510 (Tonbo). After the incubation, ~15 mL of FACS buffer was added and centrifugated at 4 °C at 300 xg for five min, supernatants were discarded, and cells were mixed with 20,000 Fluorescent AccuCheck counting beads (ThermoFisher Scientific) to calculate the number of cells in both fractions. Flow cytometry and single cell sorting was performed at the Fred Hutch Flow Cytometry Core Service using a FACSymphony S6 (BD Biosciences).

#### 2.5.2. Single cell BCR sequencing and analysis

Individual rabbit B cells from PBMC were sorted into individual wells of 96-well PCR plates (Eppendorf) using a BDSymphony S6 cell sorter following tetramer enrichment and cell surface marker staining as described above. Tp0751_24–237_ tetramer-binding B cells were sorted using side scatter height/side scatter width-based duplet discrimination, followed by gating on Ghost Dye violet 510^−^ Ig^+^ PE650^−^ APC755^−^ Tp0751_24–237_ tetramer PE^+^ Tp0751_24–237_ tetramer APC^+^ IgM^−^. Gates were set for Ghost Dye Violet 510, Ig, and IgM using fluorescence minus one (FMO) controls. After sorting, plates were sealed with adhesive PCR plate seals (ThermoFisher Scientific), centrifuged briefly and immediately frozen on dry ice before storage at −80 °C.

A nested RT-PCR approach was used to sequence paired heavy and light chain genes as described with minor modifications [23]. Reverse transcription was performed using SuperScript IV (ThermoFisher Scientific). Briefly, 7 μL of reverse transcription reaction mix consisting of 0.75 μL of 50 μM random hexamers (ThermoFisher Scientific), 0.4 μL of 25 mM dNTPs (ThermoFisher Scientific), 0.25 μL of 10 U SuperScript IV RT, 0.125 μL of RNaseOUT (ThermoFisher Scientific), 0.16 μL of 10 % Igepal (MilliporeSigma), 0.31 μL of DTT (ThermoFisher Scientific), 1.25 μL of 5× RT Buffer (ThermoFisher Scientific), and 3.61 μL of UltraPure DEPC-treated water (ThermoFisher Scientific) was added to each well containing a single-sorted B cell in 7 μL lysis buffer and incubated at 50 °C for 1 h.

Following reverse transcription, two rounds of nested PCR were conducted for both heavy and light chain using primers listed in Supplemental Table 1. For the first round, 2 μL of cDNA was added to 9.50 μL of PCR mix containing 0.1 μL 0.5 U HotStarTaq Polymerase (Qiagen), 0.0192 μL of 100 μM first round forward primer, 0.0192 μL of 100 μM first round reverse primers, 0.12 μL 25 mM dNTPs, 0.95 μL of 10× buffer (Promega), and 8.25 μL UltraPure DEPC-treated water. The first round PCR program for IgH and IgK was 50 cycles of 94 °C for 30 s, 50 °C for 30 s, and 72 °C for 55 s, followed by 72 °C for 10 min. After the first round of PCR, 2 μL of the PCR product was added to 0.1 μL 0.5 U HotStarTaq Polymerase, 0.0385 μL of each 100 μM second round forward primer, 0.0385 μL of each 100 μM second round reverse primer, 0.12 μL of 25 mM dNTPs, 0.95 μL of 10× reaction Buffer, and 8.2 μL of UltraPure DEPC-treated water. The second round PCR program was 50 cycles of 94 °C for 30 s, 55 °C for 30 s, and 70 °C for 55 s, followed by 72 °C for 10 min.

Five microliters of the PCR product were run on an agarose gel to confirm the presence of an ~500-bp heavy chain band or 450-bp light chain band. The rest of the PCR product was purified using MinElute PCR purification kit (Qiagen) and sequenced by Genewiz using the respective reverse primers. Resulting sequences were analyzed using IMGT/V-Quest to identify V, D, and J gene segments and sequences with less than 85 % V gene similarity were excluded from further analysis. Ten paired heavy and light chain sequences were produced and purified as rabbit IgG by Sino Biological.

#### 2.5.3. Antibody binding analysis

Bio-Layer Interferometry (BLI) was performed using the OctetRed (ForteBio) at room temperature with shaking at 500 RPM following the manufacturer’s instructions. Briefly, 40 μg/mL of cloned Abs were loaded on Protein A capture biosensors (Sartorius) for 240 s, followed by a wash step in 1xDPBS for 60 s. After washing, sensors were incubated in 0.1, 0.7, or 6 μM of Tp0751_24–237_ for a 5 min association stage, followed by dissociation in kinetics buffer for an additional 5 min. For display, the nm shift in signal above the baseline signal detected after antibody loading is shown.

ELISA was performed as described previously [10] with minor modifications. Briefly, 96-well polystyrene plates were incubated overnight at 4 °C with 15 pmol/well of Tp0751 in 100 μL 1xTBS, pH 7.4. Wells were blocked with 200 μL/well of 4 % skim milk powder in 1X TBS (block buffer) for 2.5 h at room temperature. Blocking buffer was removed and wells were washed four times with 1X TBS, 0.2 % Tween-20, prior to primary incubation with 50 μL/well of 0.2 pM-33 nM rabbit antibodies serially diluted in block buffer at room temperature for 1 h. The wash step was repeated, and wells were incubated at room temperature for 1 h with 50 μL/well of goat anti-rabbit IgG (H + L)-horse-radish peroxidase (MilliporeSigma) secondary antibody diluted 1:1000 in block buffer. After a final wash step, plates were developed using 3,3′,5,5′-tetramethylbenzidine (TMB) substrate system (Mandel Scientific) and read at an optical density at 600 nm using a BioTek Synergy HT plate reader (BioTek, Ontario, Canada).

#### 2.5.4. Antibody attachment inhibition assay and quantitative PCR (qPCR)

Ninety-six-well tissue culture plates (Corning, Tewksbury, MA, USA) were coated with 20,000 cells per well of the human cerebral microvascular endothelial cell line hCMEC/D3 (Millipore, Etobicoke, Ontario, Canada) and grown for 24 h at 37 °C in 5 % CO_2_ to form confluent monolayers. A total of 2-3 × 10^6^ *T. pallidum* subspecies *pallidum* (Nichols strain) in a final volume of 100 μL was harvested from *in vitro* cultures using trypsin-free dissociation media [24], and incubated for 3 h at 34 °C in 1.5 % O_2_ with 5 % CO_2_ balanced with N_2_ with either: (1) 0.25 μg of rabbit monoclonal IgG antibodies; (2) 50 μL of pooled serum from uninfected control rabbits (Gibco); or (3) 50 μL of pooled serum collected from two rabbits 120 days post-*T. pallidum* infection. Following the incubation, the serum/antibody-*T. pallidum* mixtures were added to confluent endothelial monolayers and incubated for an additional hour at 34 °C in 1.5 % O_2_ and 5 % CO_2_, balanced with N_2_. Each well was washed 3 times with 0.9 % NaCl using a BioTek Elx405 microplate washer (BioTek, Nepean, Ontario, Canada) and then lysed in buffer containing 10 mM Tris (pH 8.0), 0.1 M EDTA, and 0.05 % SDS. Lysed samples were treated with Proteinase K (Qiagen, Valencia, CA, USA) for 10 min at 56 °C and extracted using the DNeasy 96 blood and tissue kit (Qiagen), following the manufacturer’s instructions. Quantification of *T. pallidum flaA* per μg of human *GAPDH* genomic DNA was performed using quantitative PCR as previously described^7^. The % inhibition of *T. pallidum* attachment was calculated for each antibody as the percent reduction in *flaA* per μg of *GAPDH* compared to control antibody.

#### 2.5.5. AlphaFold antibody-Tp0751 structural modeling

Structural modeling of antibody-Tp0751 complexes was performed using AlphaFold 3 (www.alphafoldserver.com) [25]. The amino acid sequences of the heavy chain, light chain and Tp0751_24–237_ were submitted to the alphafoldserver.com. ChimeraX v1.8 was used for visualization and labelling of the complexes.

#### 2.5.6. Data analysis of figure generation

Data analysis and display was conducted using Excel (Microsoft), Flowjo 10 (Becton Dickinson & Company), or Prism 9 (Dotmatics) and figures compiled and refined using Illustrator 2023 (Adobe).

## 3. Results

### 3.1. Identification of Tp0751-specific murine B cells

**Fig. 1.**
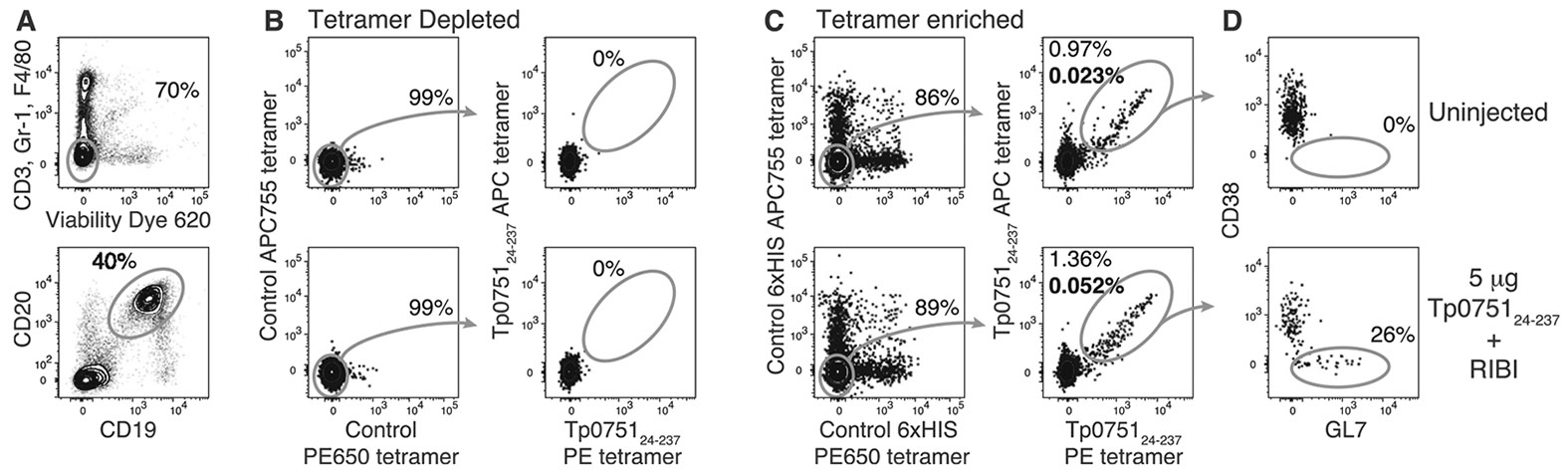
Identification of Tp0751 vaccine-responsive mouse B cells using Tp0751_24–237_ tetramers. Representative flow cytometric analysis of (**A**) live CD19^+^ B220^+^ CD3^−^ Gr-1^−^ F4/80^−^ B cells that bind Tp0751_24–237_ PE and APC tetramers but not control 6xHIS PE650 and APC755 tetramers in fractions from pooled spleen and lymph node cell samples from C57BL/6 mice (**B**) depleted or (**C**) enriched for tetramer-binding B cells using microbeads specific for PE and APC prior to analysis. The control PE650 and APC755 tetramers are included to gate out B cells specific for streptavidin, APC, PE and 6xHIS tag [16,21]. **D.** Representative flow cytometric analysis of CD38 expression and GL7 binding to gated Tp0751_24–237_ tetramer-binding B cells. Samples are representative of 2 independent experiments with mice injected subcutaneously seven days prior with 5 μg of Tp0751_24–237_ in RIBI-like adjuvant and uninjected control mice.

To identify Tp0751-specific B cells, we created fluorescent and tetrameric versions of recombinant Tp0751 encompassing amino acids 24–237, which corresponds to the mature version of the protein lacking the signal sequence. This was done by mixing biotinylated recombinant Tp0751_24–237_ with streptavidin-R-phycoerythrin (PE) or streptavidin-allophycocyanin (APC) at a ratio that fully saturates the four biotinbinding sites on streptavidin. To validate our approach, we assessed the Tp0751_24–237_ tetramer-binding B cell population in pooled spleen and lymph node samples from C57BL/6 mice injected subcutaneously with 5 μg of Tp0751_24–237_ in a RIBI-like adjuvant or uninjected controls. To ensure high specificity and sensitivity of our approach, cells were co-stained with control PE-DyLight650 (PE650) and APC-DyLight 755 (APC755) tetramers and enriched for tetramer-binding B cells using anti-PE or anti-APC microbeads. Using these approaches, a small population of B cells binding Tp0751_24–237_ tetramers but not control tetramers could be found in samples enriched for tetramer-binding cells in uninjected control animals (Fig. 1A-C). In contrast, the Tp0751_24–237_ tetramer-binding B cell population exhibited a small expansion when mice were analyzed seven days after subcutaneous injection of Tp0751_24–237_ in RIBI-like adjuvant (Fig. 1C). The expanded Tp0751_24–237_ tetramer-binding population also contained cells with downregulated expression of CD38 and increased binding of GL7 (Fig. 1D), which corresponds to a germinal center phenotype [26-28]. Together, these data indicate our ability to detect Tp0751_24–237_-specific B cell responses following Tp0751_24–237_ immunization.

### 3.2. Expansion of Tp0751_24–237_-binding B cells in immunized rabbits

**Fig. 2.**
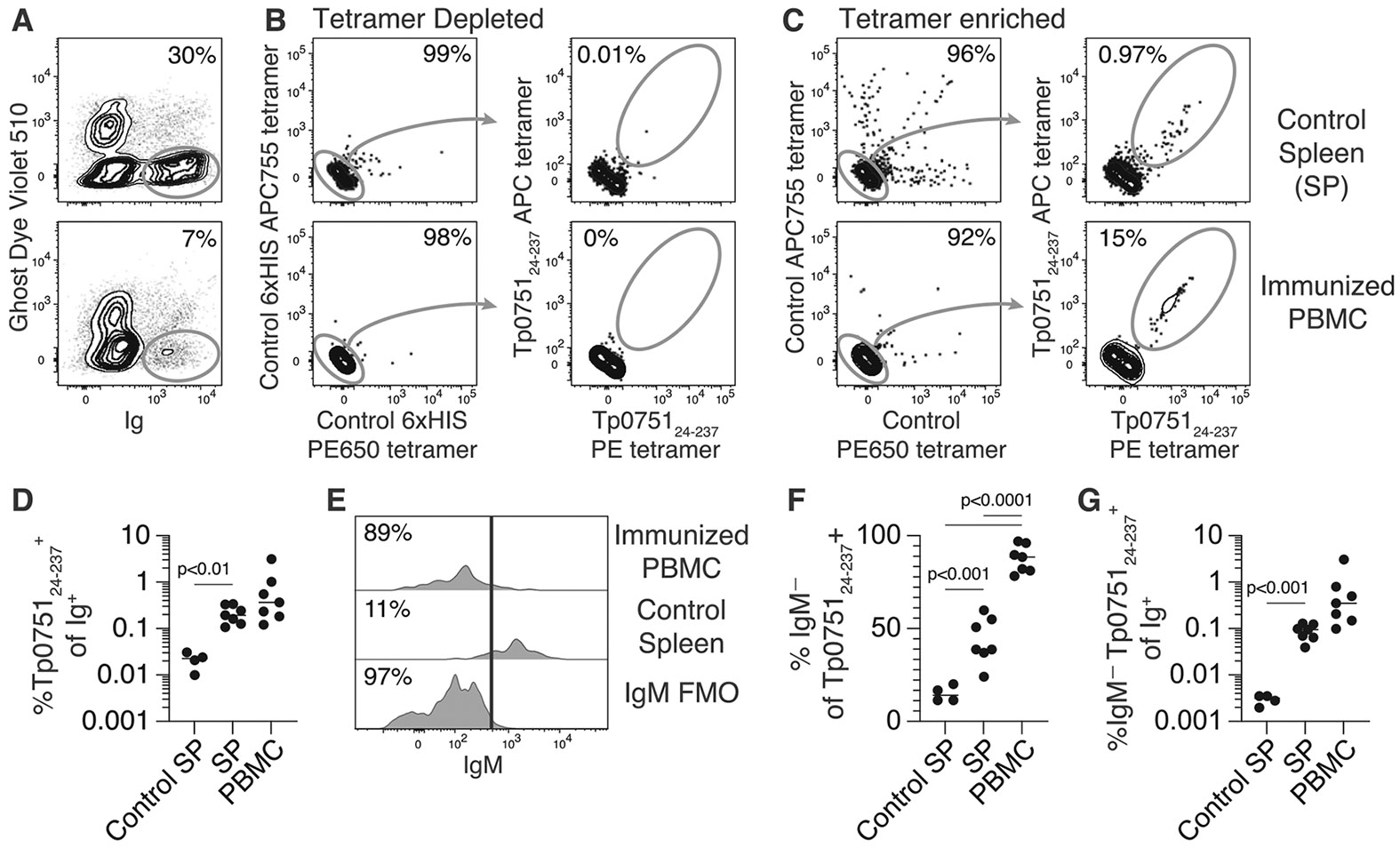
Identification of Tp0751 vaccine-responsive rabbit B cells using Tp0751_24–237_ tetramers. Representative flow cytometric gating of (**A**) live Ig^+^ cells that bind Tp0751_24–237_ PE and APC tetramers but not control 6xHIS PE650 and APC755 tetramers in fractions from PBMC from immunized rabbits or spleen cells from control rabbits (**B**) depleted or (**C**) enriched for tetramer-binding B cells using microbeads specific for PE and APC prior to analysis. **D.** Combined data from three experiments showing the frequency of Tp0751_24–237_ tetramer-binding cells amongst the total Ig^+^ population for individual rabbits in PBMC seven days after the final immunization, and in the spleen of immunized and control rabbits forty days after *T. pallidum* challenge. **E.** Representative flow cytometric gating of IgM^−^ Tp0751_24–237_ tetramer-binding B cells using an FMO control. Of note, cells were stained with antibodies specific for IgM, CD4, and CD8 in the same fluorescent channel but only IgM was left out of the FMO to exclude non-B cells expressing CD8 or CD4 from the population of interest. **F.** Combined data from three experiments showing the frequency of Tp0751_24–237_ tetramer-binding cells that were IgM^−^ in individual rabbits. **G.** Combined data from three experiments showing the frequency of IgM^−^ Tp0751_24–237_ tetramer-binding cells amongst the total Ig^+^ population in individual rabbits. In **D, F**, and **G** the lines represent the mean and *p* values were generated using a two-tailed Student’s *t*-test.

Syphilis vaccine studies are typically conducted in rabbits since mice do not exhibit symptoms similar to humans following *T. pallidum* infection [2]. Rabbits were immunized with Tp0751_24–237_ as part of a tri-antigen vaccine cocktail with two other *T. pallidum* surface antigens (TprK and TprC) in combination with RIBI-like adjuvant, as reported previously [12,13,29,30]. PBMC were collected one week following the 6th and final immunization, and two weeks later, animals were challenged with a total of 10^6^ *T. pallidum* subsp. *pallidum*, Nichols strain, per rabbit. Forty days after challenge, splenocytes were collected for analysis of B cell responses. PBMC and splenocyte samples were co-stained with Tp0751_24–237_ and control tetramers prior to enrichment using microbeads and analyzed by flow cytometry. Assessment of immunoglobulin (Ig)-expressing cells from blood and spleen revealed an increased percentage of cells binding to Tp0751_24–237_ tetramers in animals immunized with a vaccine cocktail including Tp0751_24–237_ compared to spleens from rabbits that were unimmunized and similarly challenged with *T. pallidum* (Fig. 2B-C). This expansion was largely found within cells that did not express IgM (Fig. 2D-G), suggesting that immunization with Tp0751_24–237_ induced robust isotype class switching that was absent with *T. pallidum* challenge alone. This result correlates with a prior investigation showing the limited immune response raised against Tp0751 during natural infection [31,32]. Amongst the samples, the frequency of Tp0751_24–237_ tetramer-binding cells was highest amongst PBMC from rabbit 209 (Supplemental Table 2).

### 3.3. Isolation and characterization of Tp0751_24–237_-specific rabbit antibodies

**Fig. 3.**
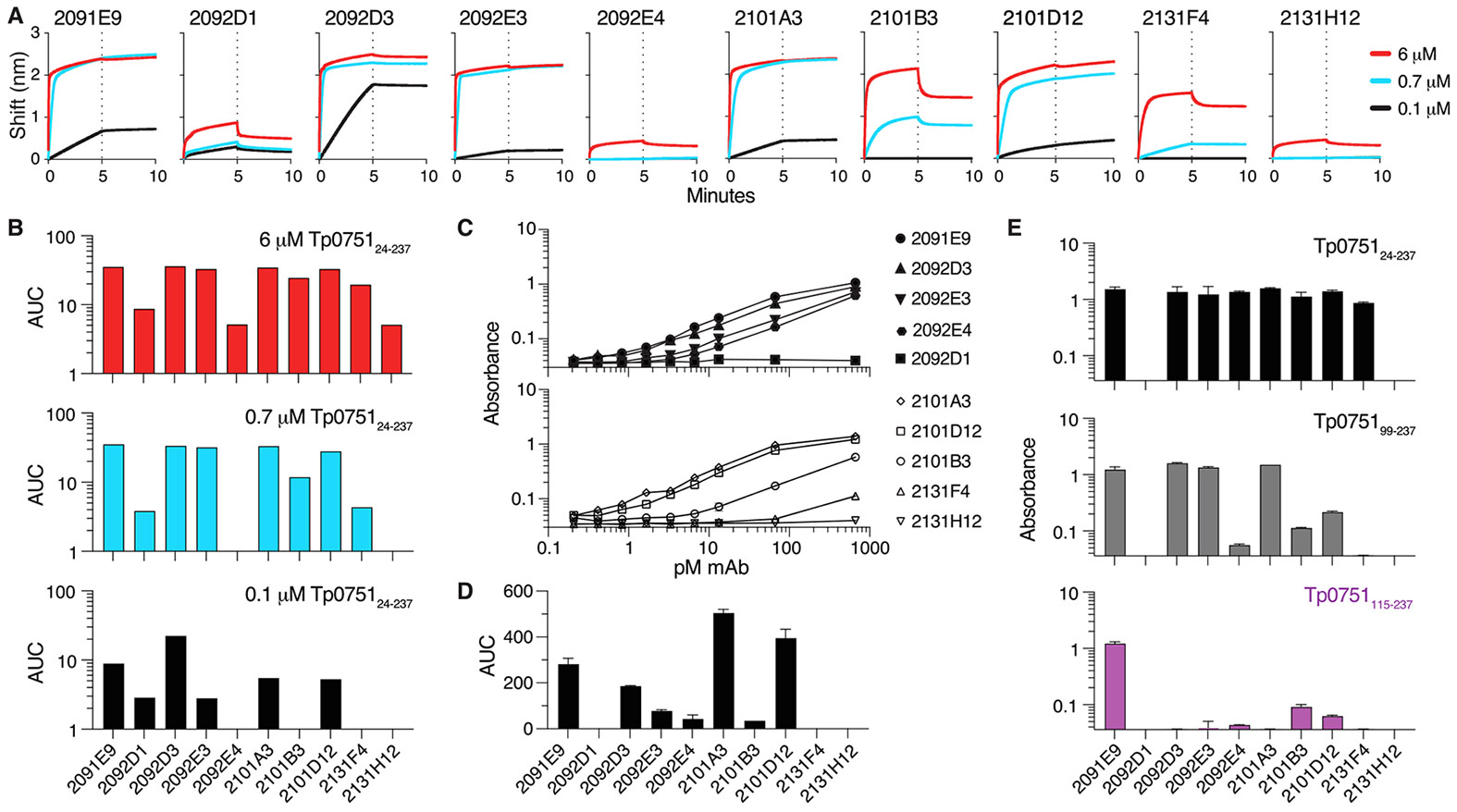
Analysis of Tp0751-specific monoclonal antibodies and mapping onto the structural regions of Tp0751. Antibodies from ten Tp0751_24–237_ tetramer-binding rabbit B cells produced as secreted rabbit IgG were assessed for binding to 0.1, 0.7, and 6 μM of Tp0751_24–237_ using Bio-Layer Interferometry (BLI) displayed as the (**A**) nm shift over time and the (**B**) area under the curve (AUC). Data representative of two similar experiments. Listed concentrations of Tp0751_24–237_-specific antibodies were assessed for binding to Tp0751_24–237_ by ELISA and displayed as the (**C**) absorbance or (**D**) AUC. Data representative of two similar experiments. (**E**) 33pM of Tp0751_24–237_-specific antibodies were assessed for binding to Tp0751_24–237_, Tp0751_99–237_, or Tp0751_115–237_ using ELISA. Data representative of two similar experiments. The bars and data points in Panels D and E represent the mean ± SEM of two technical replicates.

Next, we isolated single IgM^−^ Tp0751_24–237_ tetramer-binding B cells from the blood of immunized animals and sequenced paired heavy and light chain sequences using RT-PCR. From this, we obtained ten paired heavy and light chain sequences (Supplemental Table 3). Half of these antibodies were derived from rabbits 210 and 213, which exhibited inhibition of *T. pallidum* dissemination using the Rabbit Infectivity Test (RIT) (Supplemental Table 4). The remaining five antibodies were derived from rabbit 209, which exhibited partial inhibition of *T. pallidum* dissemination (Supplemental Table 4). All ten antibodies were confirmed to bind Tp0751_24–237_ using BioLayer Interferometry (BLI) (Fig. 3A). Amongst the cloned antibodies, 2092E4 and 2131H12 exhibited weak binding only detectable when high levels of Tp0751_24–237_ were used in the assay. Amongst the remaining eight antibodies there was variability in the association during the first five minutes of the assay, and five antibodies (2091E9, 2092D3, 2092E3, 2101A3, and 2101D12) exhibited stable binding to the point that the dissociation was not detected in the final 5 min (Fig. 3A). Strong binding of 2091E9, 2092D3, 2101A3, and 2101D12 to Tp0751_24–237_ was confirmed using ELISA-based titration assays (Fig. 3C-D).

We next used ELISA-based epitope mapping assays to determine the structural region(s) of Tp0751 targeted by each antibody. In this experiment we assessed binding to the mature Tp0751 protein (Tp0751_24–237_, comprising the full sequence minus the signal sequence) and to versions of Tp0751 produced with successive N-terminal truncations (Tp0751_99–237_ and Tp0751_115–237_). These experiments revealed that only 2091E9 showed a high level of binding to Tp0751_115–237_ (Fig. 3E). Interestingly, this region of Tp0751 consists of a lipocalin structural fold that has been shown to mediate *T. pallidum* attachment to endothelial cells and is hypothesized to play a role in traversal of *T. pallidum* across endothelial barriers and subsequent treponemal dissemination by binding the host endothelial receptor LamR [7,33]. Of the remaining seven antibodies with detectable Tp0751 binding by ELISA-based epitope mapping assays, three (2092D3, 2092E3, and 2101A3) exhibited binding to Tp0751_99–237_ but not Tp0751_115–237_, suggesting binding to the region between amino acids 99 and 114. Antibodies 2101B3, 2101D12, and 2092E4 exhibited successively reduced binding to Tp0751_99–237_ and Tp0751_115–237_ (Fig. 3E). The final antibody, 2131F4, only bound Tp0751_24–237_ (Fig. 3E). Collectively this data suggests that one antibody (2131F4) likely binds to the disordered region of Tp0751 found between amino acids 24–98, three antibodies target the alpha helix cap located between residues 99–114 of Tp0751 (2092D3, 2092E3, and 2101A3), and the four remaining antibodies appear to target an epitope entirely (2091E9) or partially (2092E4, 2101B3, and 2101D12) located within the Tp0751 lipocalin structural/functional domain [33].

### 3.4. Mapping of the Tp0751-specific antibody reactivity onto the Tp0751 structure

**Fig. 4.**
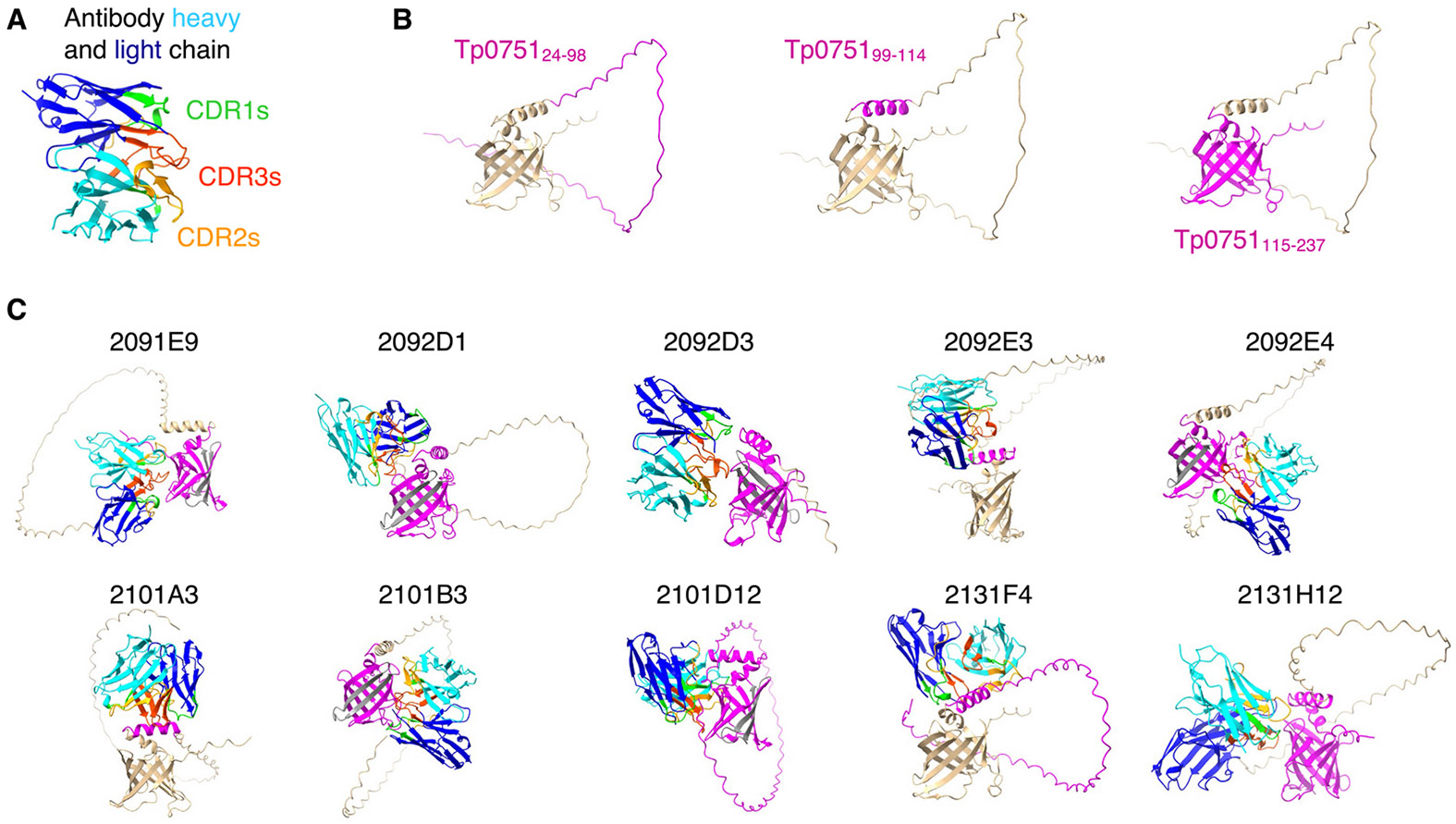
Modeling of monoclonal antibody binding to Tp0751. **A.** Predicted structure of a representative rabbit antibody fragment displaying heavy chain VDJ and light chain VJ segments. **B.** Structures of Tp0751_24–237_ [33] with different domains highlighted in magenta. **C**. Antibody:Tp0751_24–237_ complexes were modeled using AlphaFold 3. The predicted antibody binding region on Tp0751 is in magenta and the LamR binding region of Tp0751 [7] is in gray.

To extend the findings from the epitope mapping assays, we next used the antibody sequences (Supplementary Table 3) and AlphaFold 3 [25] to model the binding of the ten Tp0751-specific monoclonal antibodies with the solved structure of Tp0751_24–237_ [33] (Fig. 4). Analysis of the confidence scores indicated that most of the models surpassed confidence thresholds (Supplemental Table 5). AlphaFold modeling confirmed the ELISA-based epitope mapping results for the majority of the antibodies, with modeling showing antibodies 2092E3 and 2101A3 binding to Tp0751_99–114_, antibody 2131F4 binding to Tp0751_24–98_ and Tp0751_99–114_, antibody 2101D12 binding to all structural regions of Tp0751, antibody 2092D3 binding to Tp0751_99–114_ and Tp0751_115–237_, and antibodies 2091E9, 2092E4, and 2101B3 binding to Tp0751_115–237_ (Table 1). While ELISA-based epitope mapping studies were unable to determine the structural regions targeted by antibodies 2092D1 and 2131H12, modeling enabled prediction of the localization of antibody binding to Tp0751_99–114_ and Tp0751_115–237_. Collectively these results demonstrate how structural modeling of antibody-antigen pairs can be used to inform experimental domain mapping studies in the absence of direct visualization of these interactions using techniques like X-ray crystallography or Cryo-EM.

### 3.5. Inhibition of T. pallidum attachment to endothelial cells by Tp0751_24–237_-specific rabbit antibodies

**Fig. 5.**
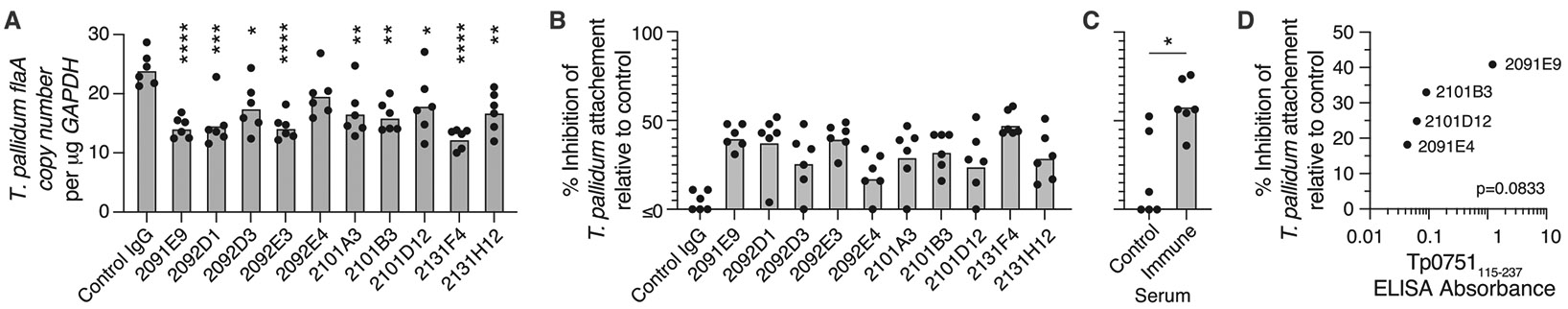
Assessment of the ability of Tp0751-specific monoclonal antibodies to inhibit *T. pallidum* attachment to human endothelial cells. The effect of monoclonal antibodies (0.25 μg) on attachment of *T. pallidum* (2-3 × 10^6^) to hCMEC/d3 cells was tested using an *in vitro* attachment assay followed by quantitation using qPCR. **A.** Attachment of *T. pallidum* to hCMEC/d3 cells was significantly inhibited by nine monoclonal antibodies. *T. pallidum* (*flaA*) copy number was normalized to human *GAPDH* from triplicate wells in two independent experiments. Bars represent means and significant differences from control antibody was analyzed using a one-way ANOVA. *p < 0.05, **p < 0.01, ***p < 0.001, ****p < 0.0001. **B.** The percent inhibition of *T. pallidum* attachment was calculated for each antibody as the percent reduction in *flaA* copy number per μg of *GAPDH* from panel A compared to control antibody. **C**. Percent inhibition of *T. pallidum* attachment using 50 μL of pooled serum collected from two rabbits 120 days post-*T. pallidum* infection compared to control serum from uninfected rabbits. Data points represent technical replicates pooled from two independent experiments. Bars represent means and *p < 0.05 calculated using an unpaired two-tailed Welch’s t-test. D. The average percent inhibition of *T. pallidum* attachment displayed *versus* the average level of detectable Tp0751_115–237_ binding detected using ELISA from Fig. 3E. The *p* value was calculated using a non-parametric Spearman correlation test.

We next assessed the ability of the isolated Tp0751_24–237_-specific antibodies to interfere with attachment of viable *T. pallidum* to a human brain microvascular endothelial cell line called hCMEC/d3. For this *T. pallidum* was incubated with hCMEC/d3 cells in the presence of Tp0751-specific antibodies or a control rabbit monoclonal antibody specific for SARS-CoV-2. Unattached *T. pallidum* were washed from hCMEC/d3 cells prior to the quantification of *T. pallidum flaA* relative to the level of human *GAPDH* genomic DNA. In these experiments, the presence of all but one Tp0751-specific antibody significantly reduced the detection of *T. pallidum flaA*, indicating reduced attachment of *T. pallidum* to hCMEC/d3 cells compared to the control antibody (Fig. 5A, B). While *T. pallidum* attachment was not eliminated in these experiments, the level of inhibition was similar to the level that could be achieved using pooled serum from rabbits previously infected with *T. pallidum* (Fig. 5B, C). Intriguingly, amongst the four antibodies exhibiting Tp0751_115–237_ binding detected by ELISA (Fig. 3E), there was a trend between the level of ELISA binding and the level of attachment inhibition that approached statistical significance (Fig. 5D). Together, these data indicate that antibodies induced by Tp0751 immunization have the potential to inhibit *T. pallidum* attachment to endothelial cells.

## 4. Discussion

In this study we characterized antibodies specific for a syphilis vaccine candidate that in prior studies has shown promising results at inhibiting bacterial dissemination in the host [2,6]. To our knowledge this study represents the first reported isolation of monoclonal antibodies from immunized rabbits that are specific for a *T. pallidum* protein that is currently under consideration for syphilis vaccine development. We identified ten rabbit-derived monoclonal antibodies able to bind to Tp0751_24–237_ tetramers used for antibody enrichment and isolation. Upon closer investigation using Tp0751 fragments that mimic the natural protein structural conformation, including fragments encompassing residues 24–237, 99–237 and 115–237, eight of these antibodies were demonstrated to bind to Tp0751 by ELISA, with selective binding to the different Tp0751 fragments. Further investigation of the specificity of the Tp0751-specific monoclonal antibodies was conducted by modeling of the antibody sequences onto the solved Tp0751 structure [33], an analysis that provided determination of the binding specificity of the two additional antibodies. Of note, both ELISA-based epitope mapping and AlphaFold modeling demonstrated four of the antibodies target the lipocalin structural fold of Tp0751_115–237_ [33]. Of these, ELISA analysis showed antibody 2091E9 had the highest level of binding to Tp0751_115–237_, and the rabbit from which this antibody was cloned demonstrated the highest frequency of B cells that bound to Tp0751_24–237_ tetramers. All ten Tp0751-specific antibodies inhibited *T. pallidum* attachment to endothelial cells, with nine achieving statistically significant levels of inhibition compared to that conferred in the presence of an irrelevant monoclonal antibody. Inhibition of *T. pallidum* attachment to endothelial cells by antibodies that bind the lipocalin structural domain of Tp0751 (residues 115–237; antibodies 2091E9, 2092E4, 2101B3, and 2101D12) aligns with the previously shown contribution of this Tp0751 structural domain to endothelial attachment [7].

We were initially surprised by the inhibition of *T. pallidum* attachment to endothelial cells mediated by the remaining six antibodies that partially or wholly target regions of Tp0751 external to the lipocalin domain. Of interest, modeling predicted that none of the ten cloned antibodies target the region within Tp0751_115–237_ that has been previously shown to mediate binding to the endothelial LamR receptor [7], a known target of neuroinvasive pathogens [34]. Based on the AlphaFold 3 models, we speculate that these six antibodies are likely to have conferred inhibition of endothelial binding via steric hinderance. Inhibition of attachment was particularly surprising for 2092D1, 2092E4, and 2131H12, which bound weakly to Tp0751 when assessed by both BLI and ELISA (Fig. 3A-B). This data likely means that the affinity by which antibodies bind to recombinantly produced Tp0751 may not reflect the potency by which antibodies bind to Tp0751 expressed by *T. pallidum*. To date, we have been unsuccessful in detecting the binding of any of ten Tp0751-specific antibodies characterized here to *T. pallidum via* immunofluorescence assays. We speculate that our inability to detect this binding is related to the low level of expression of Tp0751 on the surface of *T. pallidum*, which is common for *T. pallidum* outer membrane proteins.

Potential limitations related to this study include the lack of isolation of antibodies from rabbits that demonstrated no protection from infection, precluding the incorporation of more closely aligned comparator antibodies in the conducted investigations. Further, the precise amino acids of Tp0751 bound by each of the antibodies were not identified in this study. Future work is aimed at further characterizing the exact binding and potency of these antibodies and the identification of additional attachment-inhibiting antibodies so that the targeted epitopes can be finely mapped. This study has, however, facilitated the development of tools supporting *T. pallidum*-specific rabbit monoclonal antibody discovery, a significant knowledge gap in the *T. pallidum* field that can now be addressed. This study has also demonstrated the feasibility of protective monoclonal antibody identification in the pipeline of syphilis vaccine development.

## Supplementary Material

supplementary material

Supplementary data to this article can be found online at https://doi.org/10.1016/j.vaccine.2025.126804.

## Figures and Tables

**Table 1:** Summary of the predicted domains of Tp0751 bound by Tp0751-specific monoclonal antibodies based on antibody:antigen structural modeling performed using AlphaFold 3 compared to ELISA results.

	24–98		99–114		115–237	
			
Antibody	ELISA	AlphaFold	ELISA	AlphaFold	ELISA	AlphaFold
2091E9					✓	✓
2092D1						✓
2092D3			✓	✓		✓
2092E3			✓	✓		
2092E4	✓		✓		✓	✓
2101A3			✓	✓		
2101B3	✓		✓		✓	✓
2101D12	✓	✓	✓	✓	✓	✓
2131F4	✓	✓		✓		
2131H12				✓		✓

## Data Availability

Data will be made available on request.

## Abbreviations:

APC: Allophycocyanin
APC755: Allophycocyanin-DyLight755
AUC: Area Under the Curve
BLI: BioLayer Interferometry
FMO: Fluorescence Minus One
Ig: Immunoglobulin
PE: R-Phycoerythryn
PE650: R-Phycoerythryn-DyLight650
PBMC: Peripheral Blood Mononuclear Cells
RIT: Rabbit Infectivity Test
